# Systematic Search and Scoping Review of Physicians’ Intolerance of Uncertainty and Medical Decision-Making Uncertainties During the COVID-19 Pandemic: A Summary of the Literature and Directions for Future Research

**DOI:** 10.1007/s10880-023-09974-0

**Published:** 2023-11-06

**Authors:** Helmut Appel, Samineh Sanatkar

**Affiliations:** 1 Clinical Psychology and Psychotherapy, University of Cologne, Cologne, Germany; 2 Black Dog Institute, UNSW Sydney, Sydney, Australia

**Keywords:** Uncertainty, Intolerance of uncertainty, Medical decision making, COVID-19

## Abstract

Pandemic-related uncertainties and intolerance of uncertainty (IU) could negatively affect physicians’ well-being and functioning, being associated with experiences of distress and problematic decision-making processes. To summarize the available quantitative and qualitative evidence of physicians’ IU and decisional uncertainty during COVID-19 and problems associated with it, a systematic search was conducted to identify all relevant articles describing physician uncertainty with regard to medical decision making and well-being in COVID-19 pandemic conditions. Medical, psychological, and preprint databases were searched. Ten articles met all eligibility criteria, with eight describing quantitative and two describing qualitative research outcomes, assessed primarily in European regions and via online surveys. Associations between IU and symptoms of poor mental health and mental health risk factors were widespread, but inconsistencies emerged. Qualitative studies emphasized decisional uncertainty as a stressor for physicians, and quantitative studies suggest it may have fostered more unproven treatment choices. While the prevalence and impact of physician uncertainty under COVID-19 conditions requires further investigation, sighting available literature indicates that IU coincided with experiences of poor mental health and, at least towards the beginning of the pandemic, with willingness to endorse unproven treatments. Efforts to reduce uncertainty-related problems for physicians seem warranted, for example, through normalizing experiences of uncertainty or reducing avoidable uncertainty through maintaining open and timely communication channels.

## Introduction

Health care systems worldwide have been facing major disruptions due to the COVID-19 pandemic. Among the many challenges, the pandemic has brought about is a less tangible threat: A great deal of uncertainty (Rettie & Daniels, 2021). Physicians were arguably among the professional groups most affected by uncertainty. They had to navigate uncertainty about adequate treatment and prognosis, while being closely exposed to the novel virus, often without clear guidelines on how to best protect their patients, themselves, and their loved ones (Haier et al., 2022a, 2022b).

Uncertainty in the medical profession has been studied extensively (Bhise et al., 2018). Han et al. (2011) provide a systematic taxonomy distinguishing between three major medical uncertainty categories, referring to probabilities (i.e., probabilities being inherently indetermined, e.g., a 50:50 chance of a treatment being effective vs. ineffective), ambiguity (i.e., due to imprecise, insufficient, or conflicting information, e.g., a 30–70% chance of a treatment being effective), and complexity (i.e., overly intricate information, e.g., a wide array of interacting moderators influencing treatment effectiveness). The taxonomy illustrates how pervasive uncertainty is in the medical profession, touching on many areas of medical practice. Reflecting the relevance of uncertainty in medical practice, psychometric measurement tools assessing physicians’ handling of uncertainty have been created (Gerrity et al., 1990). Additionally, programs have been proposed to teach medical students how to deal with uncertainty (Papanagnou et al., 2021). The trait of intolerance of uncertainty (IU; Freeston et al., 1994) in the medical profession has received scholarly attention (Strout et al., 2018). IU is an individual difference trait most commonly defined as “an individual’s dispositional incapacity to endure the aversive response triggered by the perceived absence of salient, key, or sufficient information, and sustained by the associated perception of uncertainty” (Carleton, 2016b, p. 31).

Recent theorizing has highlighted fear of the unknown as a central aspect of IU (Carleton, 2016b). The unknown is widely regarded as one of the most fundamental sources of human fear, if not the most fundamental one (Carleton, 2016a). This theoretical perspective emphasizes that IU, stemming from such a core fear, serves as a causal precursor to more specific problems derived from it. Many findings align with this viewpoint, consistently demonstrating that IU is not an isolated component of a particular mental disorder but rather a trans-diagnostic risk factor for various disorders, including depression, anxiety disorders, and obsessive–compulsive disorder (McEvoy et al., 2019). Further, reducing IU causes symptom improvements for various psychological problems (Dugas et al., 2022; Li et al., 2021).

Hillen et al. (2017) introduced a comprehensive model of uncertainty management. The model stresses uncertainty *tolerance* as the more adaptive counterpart of IU,^1^ integrating IU and other variants of uncertainty tolerance-related constructs proposed across research domains. Additionally, the model includes responses to uncertainty on the cognitive (e.g., threat appraisal) emotional (e.g., fear), and behavioral (e.g., decision making) level. Hillen and colleagues further emphasize that the extent to which uncertainty is tolerated depends on moderating factors, such as aspects of the situation or the eliciting stimulus.

There is evidence of an increase in psychological burden among doctors worldwide as a consequence of the COVID-19 pandemic (Durmuş, 2022; Shanafelt et al., 2022; Troglio da Silva & Neto, 2021). Given the central role of the unknown and uncertainty as profound sources of fear, and the relevance of IU for mental health (Carleton, 2016a), it is crucial to examine the well-being and mental health implications for a profession particularly exposed to pandemic-related uncertainty. Being dispositionally unable to tolerate uncertainty or being overwhelmed by excess uncertainty despite otherwise good tolerance (Hillen et al., 2017) could result in health professionals’ mental health decline.

One of the possible behavioral consequences of pandemic-related uncertainty exceeding uncertainty tolerance of physicians, as specified in Hillen et al.’s model, is problematic decision making. Physicians are frequently required to make decisions. This includes diagnostic decisions, choosing appropriate tests and/or treatments, and deciding how best to communicate with patients (Sox et al., 2013). Especially in a pandemic context, triage decisions may also be required (Camporesi & Mori, 2021). All sources of medical uncertainty can be involved in doctors’ decision making. Additionally, these decisions often need to be made under considerable pressure, for example, because of high urgency or because a medical error could have severe consequences. High pressure decision making under conditions of sometimes extreme uncertainty is thus seen as a major challenge in medical practice (Han et al., 2011).

General predictions of how uncertainty may interfere with decision making can be found in the literature on uncertainty-related personality traits, such as IU. IU is associated with various behavioral responses, ranging from overcompensation (e.g., overplanning, excessive reassurance seeking, or extensive rumination) to avoidance (e.g., putting off uncertain situations, Bottesi et al., 2019). Rosen et al. (2014) theorized “cognitive closure” as a possible reaction, which means resorting to any answer to avoid uncertainty (Kruglanski & Fishman, 2009). Individuals with need for cognitive closure are thereby prone to blinding out conflicting, albeit potentially important information (Dolinski et al., 2016). Focusing more specifically on medical implications, previous research found associations between traits related to difficulties tolerating uncertainty and rejection of new treatments and technologies (Hamann et al., 2013; Turner et al., 2014), which may reflect reluctance to innovate. Similar associations of such traits with clinically relevant behavioral indicators, such as ordering medical tests (potentially indicative of excessive reassurance seeking) or referring patients to specialist or more intensive treatment (potentially indicative of overcaution) have been found in some studies, although not in others (see Strout et al., 2018, for an overview).

In summary, inadequate reactions to uncertainty are potentially harmful in the medical context (Gheihman et al., 2020), both regarding physician well-being and decision making. This is exacerbated by the fact that in this pandemic, uncertainty has been extreme (Koffman et al., 2020). For this reason, medical uncertainty may be of even greater importance (Sederer, 2021), potentially creating problems irrespective of IU levels (c.f., Haier et al., 2022a, 2022b; Hillen et al., 2017) and making some researchers state concerns about health system collapse (Haier et al., 2022a, 2022b). Understanding threats to physicians’ mental health and professional functioning associated with COVID-19 is a necessary step to improve their situation and reduce undesirable consequences for the health care system in similar future scenarios. We therefore review the existing evidence to clarify the role of uncertainty in physicians’ well-being and decision making during the COVID-19 pandemic.

## Methods

The focus of this review was two-fold: First, to investigate whether IU, a trait defined by the desire to avoid uncertainty, is associated with indicators of well-being, mental health, or professional functioning during the pandemic (personality/individual factor). And second, to look at how medical decision making in general has been affected by uncertainty during the pandemic (context/situation factor).

### Search Strategy

A systematic search of the literature was carried out in September 2021 and a supplementary hand search was conducted in September 2022 to update the search. Search terms were confirmed with a librarian and contained the doctor population (doctor* OR physician* OR medic* OR clinic*), decision making (decision* OR diagnostic* OR manage* OR mistake*), and uncertainty terms (uncertain* OR indecisive* OR tolerate* OR risk* OR avers* OR fear*). We decided not to include “ambiguity” in our search terms, following the reasoning of Hillen et al. (2017). They state that uncertainty “is the overarching, superordinate construct […]. In contrast, ambiguity is the subordinate construct, one of uncertainty's principal sources […]” (p. 70). We thus considered the term uncertainty to be the broader term, which includes, but is not limited to, ambiguity. Articles considered were limited to those published in 2020 or after to capture publications of data collected during the pandemic. Databases searched were Embase, PsycINFO, Web of Science, and PubMed, the latter of which also searches the Medline database. Additionally, the preprint archives of Medxriv and Psyarxiv were searched.

### Eligibility and Data Extraction

Studies were included in the review if they (i) reported on IU or decision-making ambiguity/uncertainty, (ii) examined a doctor/physician, medical student, or clinician sample, or a sample composition in which doctors made up at least 80% of participants, (iii) and data collection took place in the COVID-19 pandemic context. All articles written in English were considered. Articles where full texts were not available were excluded. Four reviewers (the two authors, HA and SS, and two research assistants) first inspected publication titles and abstracts to exclude all articles irrelevant to the review question and then verified the relevance of the remaining publications using a full text screen. Conflicting categorizations were discussed between the two authors to agree on the final selection of included articles. Resulting data were extracted into a table listing article, sample characteristics, and study results (Table 1).

**Table 1:** Overview of reviewed studies

Author, year, location	Sample demographics (*N*, *M*_*age*_, % female), professional characteristics (role, specialty)	Data collection period, recruiting procedure, study setting	Study aims and objectives	Key variables (construct: measure)	Main results related to IU or decision uncertainty
Ayoub et al. (2022), Egypt	*N* = 65, *M*_*age*_ = 35.4, 65% female; physicians working in hospital setting, various roles & specialties, either involved in COVID-19 patient treatment (63.1%) or not (36.9%)	Data collection period & recruiting procedure n/a; quantitative online-study	Assess relationships between physicians’ levels of stigma, stress coping, & IU during pandemic; compare physicians involved vs. not involved in COVID-19 patient treatment	IU: IUS-12	Inhibitory IU uncorrelated with Stigma subscales of Unfair Treatment, Stopping Self from Doing Things, and Overcoming Stigma; IU and Prospective IU uncorrelated with Coping and all Stigma subscales; no difference in IU scores between physicians involved vs. not involved in COVID-19 patient treatment
				Stigma: DISC-12	Positive Treatment subscale correlated with Inhibitory IU (*r* = .31*)
				Coping: BRCS	Coping neg. correlated with Inhibitory IU (*r* = − .33**)
Di Monte et al. (2020), Italy	*N* = 102, *M*_*age*_ = 55.1, 63% female; general practitioners	Mar. 10—May 18, 2020; through snowball sampling, several GP associations, & the State Medical Board; quantitative online-study	Assess relationships between dimensions of burnout & resilience, IU, & coping styles in Italian GPs during early pandemic; identify psychological & demographic features predicting burnout	IU: IUS-12	Higher levels of Prospective IU in High Burnout group than in Low Burnout group^a^; higher levels of Inhibitory IU in High Burnout group than in Medium Risk & Low Burnout group^a^
				Burnout: MBI	Emotional Exhaustion subscale correlated with Prospective IU (*r* = .28**) & Inhibitory IU (*r* = .31**); Depersonalization subscale correlated with Prospective IU (*r* = .23*); Personal Achievement subscale neg. correlated with Prospective IU (*r* = − .27**) & Inhibitory IU (*r* = − .27**)
				Resilience: RS-14	
				Coping: CISS	
Di Trani et al. (2022), Italy	*N* = 1,009, *M*_*age*_ = 43.9, 65% female, anesthesiologists	Feb. – Mar. 2021; e-mails sent via Italian Society of Anesthesia, Analgesia, Resuscitation and Intensive Care; quantitative online-study	Assess relationships between dimensions of burnout & resilience, IU, & coping styles in Italian anesthesiologists during early pandemic; identify psychological & demographic features predicting burnout	IU: IUS-12	Prospective IU predicts Emotional Exhaustion (*β* = .17***) & Depersonalization (*β* = .10**) Burnout subscales when controlling for age, gender, Coping, & Resilience; Prospective IU no predictor of Burnout Personal Achievement subscale & Inhibitory IU no predictor of all Burnout subscales when controlling for age, gender, Coping, & Resilience
				Burnout: MBI	Emotional Exhaustion subscale correlated with Prospective IU (*r* = .33**) & Inhibitory IU (*r* = .31**); Depersonalization subscale correlated with Prospective IU (*r* = .30*) & Inhibitory IU (*r* = .31**); Personal Accomplishment subscale neg. correlated with Prospective IU (*r* = − .25**) & IUS-12 I (*r* = − .33**)
				Resilience: RS-14	
				Coping: CISS	
Florea et al. (2021), Romania	*N* = 55, *M*_*age*_ = n/a, % female n/a; general practitioners	Apr. – Sep. 2020; through University’s mailing contacts at regional GP society; quantitative online-study	Explore GP perception of telemedicine, its reimbursement, & patient user satisfaction during the pandemic	Decision confidence	Decision confidence & Comparison to in-person consultations (combined score) correlated with Acute management of pregnant patients & Surveillance of pregnant patients (combined score, *r* = .49*); Decision confidence & Comparison to in-person consultations (combined score) uncorrelated with Patient satisfaction & Patient reactions (combined score), & Reimbursement
				GP perception of telemedicine (self-developed measures)	64.3% of GPs state that diagnostic decision making confidence may be affected by telemedicine
Johns et al. (2022), UK	*N* = 346, *M*_*age*_ = n/a., 75% female; physicians with various roles & specialties (76%), final year medical students (24%)	Sep. 2020 – Jan. 2021; through UK medical & foundation schools, social media, & private contacts; quantitative online-study	Estimate prevalence of symptoms of depression, anxiety, PTSD, & burnout in UK doctors during pandemic; assess relationships between psychological flexibility, IU, & resilience with mental health outcomes	IU: IUS-12	IUS correlated with Anxiety (*r* = .44**), Depression (*r* = .35**), PTSD (*r* = .38**), Burnout Emotional Exhaustion subscale (*r* = .31**), Burnout Depersonalization subscale (*r* = .13*); IU negatively correlated with Burnout Personal Achievement (*r* = -. 18**), Psychological Flexibility (*r* = -.41**), Resilience (*r* = -. 42**); IU predicts Anxiety (*β* = .24***) & PTSD (*β* = .18***), when controlling for other variables; IU no predictor of Depression & Burnout when controlling for other variables
				Depression: PHQ-9	
				Anxiety: GAD-7	
				PTSD: PCL-5	
				Burnout: aMBI	
				Psychological Flexibility: ComPACT-SF	
				Resilience: CD-RISC-10	
Levin et al. (2022), USA	Phase 1: *N* = 592, *M*_*age*_ = n/a, 24.8% female; Phase 2: *N* = 371,* M*_*age*_ = n/a, 25.1% female; intensive care physicians, most practiced in academic setting	Phase 1: Apr.—May 2020; Phase 2: Oct. – Nov. 2020; Phase 1: e-mails sent via third party contractor; Phase 2: through e-mail addresses collected in 1^st^ survey; longitudinal quantitative online-study	Assess predictors of unproven therapy endorsement and willingness to update therapy preferences during early phase (lack of evidence) and later phase (availability of evidence) of pandemic	Endorsement of unproven treatments: willingness to treat with debated drugs in COVID-19 scenario vignette	Endorsement of unproven treatments associated with more Evidence skepticism (RR 1.33, 95% CI [1.22, 1.44]), Need for Closure (RR 1.18, 95% CI [1.05, 1.32]), Risk Tolerance (RR 1.12, 95% CI [1.04, 1.21]) when controlling for other variables; Endorsement of unproven treatments associated with less Research engagement (RR .94, 95% CI [.91, .98]) when controlling for other variables
				Resistance to update beliefs: change of treatment preferences in COVID-19 scenario vignette from 1^st^ to 2^nd^ survey in accordance with scientific evidence published in meantime	Resistance to update beliefs associated with more Evidence skepticism for antimalarials (RR 1.85, 95% CI [1.01, 3.41]) and corticosteroids (RR 2.24, 95% CI [1.45, 3.45])
				Decisional uncertainty: assumed to be included in COVID-19 scenario	
				Need for Cognition: NCS	
				Risk Tolerance: RO	
				Need for Closure: NFCS-15	
				Research engagement	
				Evidence skepticism	
Lynch et al. (2022), USA	*N* = 18, *M*_*age*_ = n/a, 61.1% female; GI medical oncologists (33.3%), gynecologic medical oncologists (33.3%), hospitalists (33.3%)	Data Collection Period = n/a; through e-mails to selected physicians; qualitative virtual interview study (via Zoom)	Qualitatively explore problems (incl. practice changes, challenges, and emotional burden) in oncologists in an epicenter of the early pandemic (NYC)	Not applicable (qualitative study using thematic analysis)	Summary of thematic patterns: Oncologists reported five COVID-19-related categories of stressors grouped around the constructs of fear and distress, respectively; ambiguity in decision making and impact of uncertainty and acuity of COVID-19 on goals-of-care discussions emerged as important stressors
Martínez-Sanz et al. (2020), Spain	*N* = 852, *M*_*age*_ = 39.0, 54% female; physicians involved in COVID-19 patient treatment, various roles & specialties	Apr. 12 – Apr. 19, 2020; through e-mails to international professional networks & Twitter; quantitative online-study	Assess predictors of therapeutic aggressiveness in COVID-19 scenario vignettes during first weeks of pandemic	Number of selected treatments in COVID-19 scenario vignettes	Internal medicine (OR 1.67, 95% CI [1.31, 2.08]) & pneumology (OR 1.98, 95% CI [1.13, 3.46]) associated with higher Number of selected treatments than infectologist specialization; Female gender associated with higher Number of selected treatments (OR 1.17, 95% CI [1.02, 1.33]); Practicing in North or Latin America associated with lower Number of selected treatments ^b^; First-authorship of PubMed-indexed articles associated with lower Number of selected treatments ^b^; Severity of scenario associated with higher Number of selected treatments^b^
				Self-perceived expertise in COVID-19	Higher Self-perceived expertise associated with higher Number of selected treatments (OR 1.95, 95% CI [1.31, 2.89])
				Perceived quality of COVID-19 publications	Higher Perceived quality of COVID-19 publications associated with higher Number of selected treatments (high vs. low quality, OR 1.92, 95% CI [1.17, 3.16])
Perumalswami et al. (2022), USA	*N* = 22, *M*_*age*_ = 52.0, 36% female; oncologists	Jan. – Aug. 2020; through e-mail, fax, & phone, randomly selected from groups formed according to sex, race, etc.; qualitative interview study (setting n/a)	Qualitatively explore problems & their management in oncologists during the pandemic	Not applicable (qualitative study using thematic analysis)	Summary of thematic patterns: Oncologists reported high levels of uncertainty & fear; increases in stress were managed through coping mechanism of non-abandonment; complex decision making required frequent compassionate communication through telemedicine
Salvato et al. (2021), Italy	*N* = 64, *M*_*age*_ = n/a, 50% female; physicians involved in COVID-19 patient treatment (76.6%) or not (23.4%), various roles & specialties	Apr. 10 – Apr. 30, 2020; through the Italian Medical Council network; quantitative online-study	Assess effect COV-ID-19 vs. other medical scenarios & interoceptive competence on off-label drug endorsement among physicians	Interoception: MAIA	Trusting subscale predicts Off-label drug endorsement, *b* = .36*
				Self-developed 15-item questionnaire of off-label drug endorsement	Higher Off-label drug endorsement in COVID-19 than normality scenario, *t* = -3.92**; No difference in Off-label drug endorsement between COVID-19 & other emergency scenario; Off-label drug endorsement uncorrelated with COVID-19-related variables; Off-label drug endorsement uncorrelated with Anxiety
				Decisional uncertainty: assumed to be included in COVID-19 scenario	
				Anxiety: STAI X	

*BRCS* Brief Resilient Coping Scale (Sinclair & Wallston, 2004; English/Arab translation not available); *CD-RISC-10* Conor Davidson Resilience Scale (Connor & Davidson, 2003); *CISS* Coping Inventory for Stressful Situations (Endler & Parker, 1994; English/Italian translation: Sirigatti & Stefanile, 2009); *ComPACT-SF* Comprehensive Assessment of ACT Processes (Morris, 2019); *DISC-12* Discrimination and Stigma Scale, version 12 (Brohan et al., 2013; English/Arab translation not available); *IU* Intolerance of Uncertainty; *IUS-12* Intolerance of Uncertainty Scale, 12 items version (Carleton et al., 2007; English/Italian translation: Lauriola et al., 2016; English/Arabic translation: Alatrany, 2020); *GAD-7* Generalized Anxiety Disorder Scale (Spitzer et al., 2006); *MAIA* Multidimensional Assessment of Interoceptive Awareness (Mehling et al., 2012; English/Italian translation: Calì et al., 2015); *MBI* Maslach Burnout Inventory (Maslach et al., 1997; English/Italian translation: Sirigatti & Stefanile, 1993; abbreviated version (aMBI): McManus et al., 2002); *NCS* Need for Cognition Scale (Cacioppo et al., 1984); *NFCS-15* Need for Cognitive Closure Scale, 15 items version (Roets & Van Hiel, 2011); *PCL-5* Post-traumatic Stress Disorder Checklist for DSM-5 (Blevins et al., 2015); *PHQ-9* Patient Health Questionnaire (Kroenke et al., 2001); *RO* Risk Orientation, single-item measure (Maestas & Pollock, 2010); *RS-14 * The 14-Item Resilience Scale (Wagnild & Young, 1993; English/Italian translation: Callegari et al., 2016); *STAI* State-Trait Anxiety Inventory (Spielberger et al., 1970; English/Italian translation: Pedrabissi & Santinello, 1989); *OR* Odd Ratio, *RR* Relative Risk, *95% CI* 95% Confidence Interval

^a^Only *p* value indicated

^b^Test statistics n/a

*p < .05; ** *p* < .01; *** *p* < .001

## Results

**Fig. 1:**
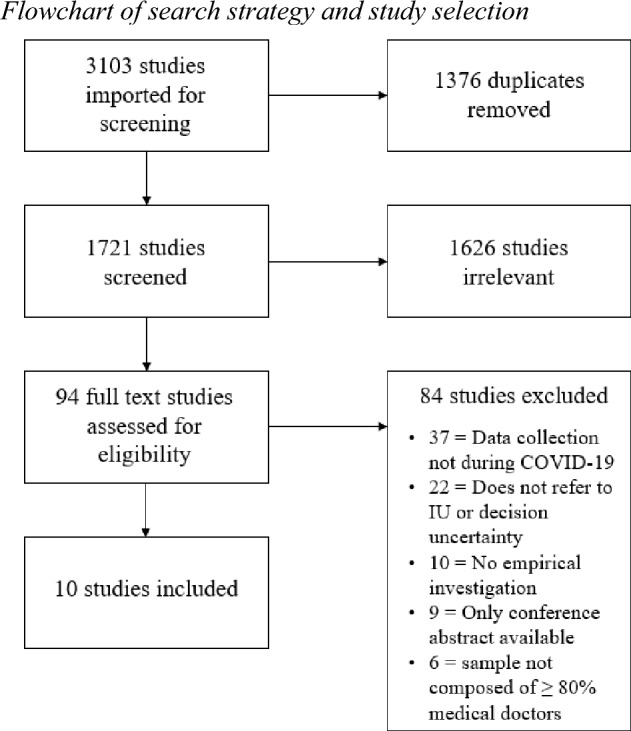
Flowchart of search strategy and study selection

The search yielded a total of 1,721 articles across databases and after removal of duplicates. A flowchart of the screening process is depicted in Fig. 1.

Ten articles met all study eligibility criteria. Data collection took place between March 2020 and March 2021 and was initiated by research groups in Eastern (Florea et al., 2021) and Southern Europe (Di Monte et al., 2020; Di Trani et al., 2022; Martínez-Sanz et al., 2020; Salvato et al., 2021), UK (Johns et al., 2022), North America (Levin et al., 2022; Lynch et al., 2022; Perumalswami et al., 2022), and Northern Africa (Ayoub et al., 2022). Five samples were comprised of physicians with different roles and specialties (Ayoub et al., 2022; Johns et al., 2022; Levin et al., 2022; Martínez-Sanz et al., 2020; Salvato et al., 2021), two samples were comprised of general practitioners (Di Monte et al., 2020; Florea et al., 2021), two samples were comprised of oncologists (Lynch et al., 2022; Perumalswami et al., 2022), and one was comprised of anesthesiologists (Di Trani et al., 2022). All but two studies (Lynch et al., 2022; Perumalswami et al., 2022) were quantitative assessments and collected data via online survey tools. Four of the quantitative assessments employed the validated Intolerance of Uncertainty Scale (IUS-12, 12 item version: Carleton et al., 2007) to measure physicians’ level of IU during the COVID-19 pandemic (Ayoub et al., 2022; Di Monte et al., 2020; Di Trani et al., 2022; Johns et al., 2022), while in one study, choosing multiple and/or non-evidence-based treatments was interpreted as a proxy for IU, measured by the number of selected treatments in COVID-19 medical scenario vignettes (Martínez-Sanz et al., 2020). Medical decision-making uncertainty, on the other hand, was operationalized in different ways. In one study, it was assessed using face valid items constructed ad hoc by the authors (Florea et al., 2021). In three other studies, decisional uncertainty was included in the stimulus material (Levin et al., 2022; Martínez-Sanz et al., 2020; Salvato et al., 2021). Finally, the qualitative studies exploring decisional uncertainty summarized findings by way of thematic analysis.

The aims of the studies ranged from observing medical professionals’ mental health and its antecedents, including IU, during the pandemic to investigating whether doctors’ medical decision making changed as a result of the pandemic. Table 1 provides an overview of the reviewed studies. In the following, the results will be presented as a narrative summary based on Higgins et al.’s (2022) recommendations. We grouped articles based on whether the described studies were designed to examine mental health outcomes or decision-making outcomes.

## Intolerance of Uncertainty and Mental Health Variables During COVID-19

Mental health symptoms assessed were depression, anxiety, post-traumatic stress (Johns et al., 2022), and burnout (Di Monte et al., 2020; Di Trani et al., 2022; Johns et al., 2022).^2^ Standardized measures of mental health antecedents (i.e., risk and protective factors)—besides IU—included resilience (Di Monte et al., 2020; Di Trani et al., 2022; Johns et al., 2022), coping (Ayoub et al., 2022; Di Monte et al., 2020; Di Trani et al., 2022), mental health stigma (Ayoub et al., 2022), and psychological flexibility (Johns et al., 2022). When looking at bivariate associations, IU showed weak to moderate positive associations (*r*s ranged between .13 and .44, *p*s < .05) with depression, anxiety, post-traumatic stress, and burnout (Di Monte et al., 2020; Di Trani et al., 2022; Johns et al., 2022). However, Johns et al. (2022) observed that statistically significant associations with depression and burnout did not persist when other variables were accounted for, in particular, age, resilience, and coping. Similarly, Di Monte et al. (2020) found that prospective IU showed no statistically significant associations with burnout subscales when controlling for resilience, coping, and demographic information. Results for inhibitory IU were not reported. In contrast, using the almost identical constellations of variables, Di Trani et al. (2022) reported that prospective IU was statistically significantly associated with the emotional exhaustion (*β* = .17, *p* < .001) and depersonalization (*β* = .10, *p* < .01) burnout subscales, but not with personal accomplishment. Inhibitory IU was not associated with burnout in Di Trani et al.’s analysis.

Regarding mental health antecedents, Johns et al. reported negative correlations between IU and resilience (*r* = − . 42, *p* < .01) and psychological flexibility (*r* = − .41, *p* < .01), while Ayoub et al. reported negative correlations between inhibitory IU and coping (*r* =  − .33, *p* < .01). However, there were no associations between prospective IU and coping. A statistically significant positive relationship was observed between inhibitory IU and the positive treatment dimension of the stigma due to treating COVID-19 patients measure (*r* = .31, *p* < .05), but not for the other IU scores, nor for the other stigma subscales (Ayoub et al., 2022).

Interestingly, using the IUS-12, Ayoub et al. (2022) observed no differences between physicians directly involved in COVID-19 patient care and those who were not. However, the IUS, being a trait measure, is not intended to detect situation-dependent changes in IU experiences.

## Uncertainty and Medical Decision Making During COVID-19

Studies looking at decision uncertainty in the medical context during the pandemic utilized different approaches. Perumalswami et al. (2022) and Lynch et al. (2022) collected qualitative reports from oncologists concerning medical decision making in general. The researchers observed that considerable levels of uncertainty prevailed in the first months of the pandemic, which caused negative feelings of fear and distress concerning medical decision making (Lynch et al., 2022; Perumalswami et al., 2022). These negative experiences of decisions were further fueled by ethical challenges like moral dilemmas (e.g., resource shortages, Perumalswami et al., 2022; or treatment delays because of infection risk, Lynch et al., 2022), or the disruption of the usual communication channels, requiring sometimes complicated technological communication aids. Participants also reported on their coping strategies, emphasizing non-abandonment. Also investigating general implications for decision making, but focusing on telemedicine due to the pandemic, Florea et al. (2021) found a majority of their sample reported negative effects of telemedicine on their decision confidence.

Looking more specifically at treatment choices, Salvato et al. (2021) included uncertainty in their stimulus material comprised of three medical scenarios (standard, emergency, COVID-19). The researchers argued that the COVID-19 scenario involved the highest degree of uncertainty and looked at endorsement of non-evidence-based off-label drug use. Results showed that physicians expressed greater legitimacy of non-evidence-based approaches in the emergency and the COVID-19 scenario compared to the standard scenario (Salvato et al., 2021). Relatedly, Levin et al. (2022) observed physicians’ endorsement of unproven therapies in a severe COVID-19 case scenario at the onset of the pandemic, thus including a high degree of uncertainty (lack of conclusive evidence). Levin et al. found high endorsement rates and, more importantly, endorsement was associated with traits hindering up-to-date evidence-based practice, such as need for cognitive closure (RR, 1.18; 95% CI [1.05, 1.32]), skepticism towards clinical evidence (RR, 1.33; 95% CI [1.22, 1.44]), and risk tolerance (RR, 1.12; 95% CI [1.04, 1.21]). However, in a follow-up survey administered in late autumn 2020, most respondents had updated their early treatment preferences in accordance with evidence published in the meantime (Levin et al., 2022).

Finally, as mentioned earlier, Martínez-Sanz et al. (2020) assessed “aggressive” (i.e., combining multiple and/or non-evidence-based treatments) versus conservative treatment choices and reported demographic and professional factors that increased the odds of endorsing a greater number of treatments in a COVID-19 scenario. The researchers found male gender and residing in North and Latin American countries to be associated with choosing fewer unproven treatments as opposed to female gender and residing in European and other countries. Specialization in infectious diseases was associated with more conservative choices when compared to physicians specialized in pneumology and internal medicine. Doctors who chose fewer unproven treatments reported being first author of scientific publications more often than those who showed greater readiness to choose these treatment options. Further, higher levels of “aggressiveness” were associated with more self-perceived COVID-19 expertise (OR 1.95; 95% CI [1.31, 2.89]) and perceived quality of COVID-19 publications (OR 1.92; 95% CI [1.17, 3.16]). Most notably, the more severe the presented COVID-19 scenario, the more unproven treatments were chosen and, across the whole sample, the more variety and less agreement in the chosen treatments were found.

## Discussion

### Summary of Results

As new COVID-19 variants with unknown risk profiles may emerge, and pandemics are expected to occur more frequently (Marani et al., 2021), uncertainty will stay a major challenge for practicing physicians (Koffman et al., 2020) and it is important to be aware of the impact of uncertainty. This review was therefore designed to assemble the available evidence on decisional uncertainty and IU among physicians during the pandemic. Despite substantial variety in methodological approaches, some patterns do emerge across the reviewed research. These patterns relate to associations between IU and physician mental health and medical decision making.

#### Relationships of IU with Mental Health Variables

Studies including the IUS-12 consistently found small-to-moderate relationships between IU and measures of mental health and mental health risk or protective factors, except stigma associated with treating COVID-19 patients. When simultaneously testing IU and other potential risk or protective factors, IU did not consistently remain a significant predictor of well-being and mental health outcomes (Di Monte et al., 2020; Johns et al., 2022). However, Di Trani et al. (2022) observed a significant positive association between (prospective) IU and burnout over and above resilience and coping, indicating that IU contributes unique variance to physicians’ experiences of burnout.

There are several possible explanations for the lack of statistically significant associations with IU in some of these studies. Fear of the unknown as the essential component of IU is a most fundamental fear (Carleton, 2016b). Consequently, IU should be considered a lower-order antecedent of the predicted dependent variables compared to the more proximal, higher order variables of coping, resilience, and psychological flexibility. It is therefore logical that IU does not explain additional variance in a simultaneous regression with these concurrent predictors. A mediation analysis with IU predicting the dependent variables indirectly through the other predictors would be the more appropriate analysis, but is not reported. This causal hierarchy could also have been mapped in a longitudinal analysis, so the purely cross-sectional analyses are not optimal.

Considering the small sample sizes, the analysis presented by Di Monte et al. could also be underpowered, especially considering the high number of tested variables (Ioannidis, 2005). The effects found by Di Trani et al., based on a large sample, support this reasoning.

Finally, all studies including IU in a multiple regression (Di Monte et al., 2020; Di Trani et al., 2022; Johns et al., 2022) used different scores of the IUS-12 and its sub-components, potentially creating diverging results. The most common distinction concerning IU sub-components is between inhibitory IU (being paralyzed by uncertainty) and prospective IU (being overly vigilant and cautious in the face of uncertainty; Carleton et al., 2007). Although there is an ongoing debate about the utility of the IUS-12 subscales (Hale et al., 2016), differential relationships between each subscale and psychopathological symptoms have been documented (see McEvoy et al., 2018, for an overview). Different results depending on which subscale was included in the analysis are therefore conceivable. For example, subscale-based analysis for Johns et al. (2022) would be informative in order to see whether prospective IU remains a statistically significant predictor when controlling for other variables, similar to Di Trani et al. (2022).

The observed negative relationships between IU and mental health converge with pre-pandemic evidence showing that, even in the absence of a highly uncertain new pandemic, IU in physicians is associated with such negative outcomes as burnout (Begin et al., 2022; Cooke et al., 2013), or low professional confidence (Lin et al., 2022). These findings are also in line with research on how IU relates to coping with or being impaired by the COVID-19 pandemic in non-physician populations. For example, in samples from the general population, IU has been associated with COVID-19-specific distress (Taylor et al., 2020) and general well-being and mental health (Rettie & Daniels, 2021; Satici et al., 2022), a pattern already found for other pandemics like H1N1 (Taha et al., 2014). Previous research further identified IU as a risk factor for psychopathology in general (McEvoy et al., 2019) and for negative affect in situations of extreme uncertainty like a pandemic (Freeston et al., 2020).

It needs to be noted that the reviewed articles do not afford causal inferences due to their correlational nature. More importantly, it is difficult to estimate whether the IU associations with various problems have changed due to the pandemic. Strictly speaking, correlations between IU and mental health variables may reflect general, pandemic-unspecific relationships (cf. Begin et al., 2022). We know of no research directly comparing pre- and post-onset of the pandemic whether relationships between IU and indicators of well-being, mental health, or professional functioning in physicians have changed. There is, however, evidence of increased mental health burden on physicians as a function of the pandemic’s severity (e.g., regarding burnout, Shanafelt et al., 2022).

#### Findings on Decisional Uncertainty

Studies examining medical decision making indicated that drastic cases of COVID-19 produced a tendency to resort to unproven treatments. Uncertainty coupled with massive pressure on health care systems, as seen in the COVID-19 pandemic, may increase the rate of non-evidence-based treatments. While this is in line with the understandable “’do something’ principle” (Martínez-Sanz et al., 2020, p. 2), such treatments may prove ineffective or even harmful (Rubin et al., 2020). Supporting this reasoning, the tendency to endorse unproven treatments is associated with traits defined by blinding out uncertainty and complexity at the expense of adhering to scientific evidence (Levin et al., 2022), such as need for closure (Kruglanski & Fishman, 2009). This suggests that handling uncertainty well is important for making desirable COVID-19-related medical decisions. The pattern of results is in line with the behavioral consequences of IU specified in the model provided by Hillen et al. (2017), predicting altered decision making as a function of how well uncertainty is tolerated. However, this interpretation must also be made with caution. The studies we identified did not directly measure or experimentally manipulate decisional uncertainty. Martínez-Sanz et al. and Salvato et al. systematically varied the severity of the COVID-19-scenario and assumed that the increased uncertainty was the driver of more non-evidence-based treatment choices. However, such treatment choices may be explained by reasons other than only uncertainty (e.g., time pressure, condition severity, etc.). Situational factors can indeed interact with uncertainty, for example, by making uncertainty less tolerable, and thus moderating the response (Reuman et al., 2015). This interaction is applicable to the medical context as well, where situational factors are also likely to influence uncertainty management (e.g., as in the decision whether to admit a patient with severe symptoms vs. in the decision whether to order a test for a patient with mild symptoms; cf., Hillen et al., 2017). However, technically speaking, the given design does not allow to disentangle potential driving factors, which would require manipulating them independently (Reuman et al., 2015). Therefore, more research is needed to substantiate the role of uncertainty in these effects.

In two qualitative studies, physicians spontaneously named decision-making uncertainty as a special challenge brought about by the pandemic. Also, in the study by Florea et al. using ad hoc-constructed items, decision uncertainty was raised by almost two thirds of GPs. While results derived from qualitative studies do not allow estimating the relative importance of decision-making uncertainty compared to other stressors, this suggests that pandemic-related decisional uncertainty was widespread and complicated physicians’ works regardless of individual vulnerability. Building on these findings, it is important to point out that physicians’ uncertainty-related problems during the pandemic should not be regarded as a personal weakness. Instead medical decision-making uncertainty is likely to arise when unknown factors accumulate (Haier et al., 2022a, 2022b) and, while there are inter-individual differences in the level of discomfort this can bring about, it should be regarded as an occupational risk factor to be anticipated and managed.

### Limitations of Reviewed Evidence and Recommendations for Future Research

The reviewed studies provided limited insight into the burden on physicians’ mental health and well-being due to IU and decision-making uncertainty during the COVID-19 pandemic. Partially, they contained methodological flaws. Below, we will therefore discuss limitations and directions for future research.

First, valid operationalizations should not be neglected. For example, Martínez-Sanz et al. (2020) used a questionable IU operationalization (i.e., choosing multiple and/or non-evidence-based treatments). However, in this context, it is unclear whether such choices are in line with the definition of IU (Carleton et al., 2016), especially as IU can have very diverse behavioral correlates (Bottesi et al., 2019) and behavior-based measures of IU have not yet been established (Jacoby et al., 2016). Using direct and established IU measures, for example “Situational IU” (Mahoney & McEvoy, 2012), could have prevented this ambiguity. In some cases, there may be no alternative to construing new items, such as for self-perceived expertise in COVID-19 treatment (Martínez-Sanz et al., 2020). However, representing an extreme example, (Florea et al., 2021) conducted an exploratory study using only ad hoc self-constructed measures. If the aim is collecting participants’ general themes and impressions, qualitative approaches offer a more thorough appreciation and less risk of erroneous inferences compared to unvalidated quantitative measures with unclear psychometric properties (e.g., Haier et al., 2022a, 2022b).

Similarly, statistical power should always be considered in quantitative research. Although some studies involved large samples affording statistical power appropriate for the conducted analyses (e.g., Di Trani et al., 2022: *N* = 1,009), only one publication included explicit statistical power considerations (Johns et al., 2022). Other studies were based on small samples around 100 participants or fewer (Ayoub et al., 2022; Di Monte et al., 2020; Florea et al., 2021; Salvato et al., 2021). Small sample sizes restrict conclusiveness, especially when effect sizes are small (da Silva Frost & Ledgerwood, 2020; de Winter et al., 2016). For example, for multiple regression, more recent publications have suggested minimum observations per predictor variable as high as *n* = 37 (Harrell, 2015) or even *n* = 100 (Brysbaert, 2019) as a rule of thumb when testing individual predictors for statistical significance in typical research scenarios. This would call into question the adequate statistical power for Di Monte et al.'s (2020) regression analyses, testing four concurrent predictors with 102 participants. Still, under certain circumstances, small samples may be the best data available given resource restrictions. In this case, simple analyses such as bivariate correlations (Ayoub et al., 2022; Florea et al., 2021) are preferable to elaborate models with large numbers of predictors (Di Monte et al., 2020; Salvato et al., 2021).

To further scientific insight, measures should be used in a way that allows comparison between studies. For example, in the reviewed studies, varying IU scores were used, including studies that only used the two subscales (Di Trani et al., 2022) or even one of the subscales (Di Monte et al., 2020) instead of the total score. Because the factor structure of the IUS-12 is still under debate and recent research has favored using a total score (Hale et al., 2016; Shihata et al., 2018), results based on the total scale should at least be reported additionally, even if hypotheses refer to the subscales.

Additionally, insight is limited by the difficulty to assess the COVID-19-specific impact on the reported effects. Naturally, the pandemic cannot be experimentally manipulated. However, at least proxies modeling the pandemic’s effect are conceivable. For example, Sauer et al. (2020) asked participants retrospective questions to compare hypochondriac safety behavior before and after the onset of the pandemic. Shanafelt et al. (2022) used a longitudinal design comparing an early and a later stage of the pandemic and related measures of burnout to different pandemic phases. Similarly, in the future or in analyses based on existing COVID-19 data, longitudinal designs could be used to compare more and less intense points of the pandemic (e.g., winter vs. summer, higher vs. lower hospitalization rates) and their direct or moderating influence on other variables and their relationships, respectively.

Finally, future studies should strive to extend geographic representativeness. For example, seven of the ten reviewed studies focused on European and US participants. South-western Europe and parts of the USA were among the regions hit hardest by the pandemic (Díaz Ramírez et al., 2022). At the same time, considering the global scale of the pandemic, other regions covering a wider cultural range should also be represented. Likewise, global collaboration between laboratories with similar research interests is called for more than ever. Undertaking concerted research using the same measures and translating them, if required, as well as unified analysis procedures would largely increase interpretability and allow meta-analytic evaluation. This would also help prevent the emergence of a vast array of different measures for the same construct, thereby hindering comparability, as has been seen, for instance, in the case of scales assessing COVID-19-related anxiety (e.g., Ahorsu et al., 2022; Lee, 2020; Silva et al., 2022). In the case of IU, the UNiCORN project (www.covid19an.com) has taken this approach in order to effectively unite forces in studying the interaction of IU and the pandemic’s effects and across different countries.

### Strengths and Limitations of Review Procedure

This review provides valuable insight into medical decision uncertainty and whether IU is a mental health risk factor in physician populations, particularly under high uncertainty circumstances. While IU has been recognized as important in the medical domain (Strout et al., 2018), its impact remains understudied, especially under pandemic circumstances. This systematic search contributed to establishing the scope of IU under COVID-19 conditions encompassing all major databases and preprint archives and complemented by hand search.

Several limitations should be noted. First, we restricted the search to English articles. Considering the global scope of the pandemic and regional hotspots outside English speaking countries, this may have caused exclusion of evidence if it was published in a different language. Second, we only identified qualitative studies in our search that matched our search terms. These specific search terms would only appear in an article if they were mentioned by participants or if authors chose those terms to describe themes. Thus, we were unable to assess how many qualitative studies on physicians’ experiences of the pandemic did *not* include IU or decisional uncertainty. We can therefore not quantify how important uncertainty is in its relative contribution to work stress and burden across qualitative assessments. Similarly, if studies using quantitative methods did not select IU as potential contributors to doctors’ experiences, this search would not detect them. Finally, some of the included findings were based on insufficient study designs or were difficult to interpret, limiting the knowledge that can be derived from this work. Future reviews may afford a broader scope of the research question and search to identify a higher number of relevant articles.

### Directions for Interventions to Reduce Intolerance of Uncertainty

The clear connection between the handling of uncertainty and well-being among physicians were known before the pandemic (Strout et al., 2018). Although interventions to improve tolerance towards uncertainty have shown promising results (Patel et al., 2022), the findings of this review suggest that the uncertainty of the pandemic still created sizeable problems. Considering the burden of impaired physician well-being not only on a personal level, but also in terms of quality of care and patient safety (Shanafelt et al., 2022), efforts to address uncertainty are urgently needed. Before mentioning interventions, a few general remarks seem appropriate. First, frontline health care professionals have not been the only ones affected by the COVID-19 pandemic and its uncertainties, but most active physicians, having to adapt constantly to changing risk estimations and regulations. Perumalswami et al. (2022) and Lynch et al. (2022) focused on oncologists, whose treatments were greatly affected even when their patients were not themselves infected, for example because of changed resource allocation. Therefore, when introducing measures, all specialization groups should be considered.

Second, although it is important to propose individual-level interventions, problems with decisional uncertainty may not be attributed exclusively to the individual, suggesting personal weakness. Likewise, targeting problems associated with IU does not mean high IU individuals are to be blamed when struggling with uncertainty. Under normal conditions, IU may even involve important professional virtues (Reis-Dennis et al., 2021). But in an extreme situation, being very exposed to COVID-19 and working under high pressure, the health care system is a difficult work environment and structural interventions are equally called for.

That being said, attitudes towards uncertainty should be addressed among physicians. Often, a high degree of certainty is demanded of physicians despite limited information and ambiguous evidence (Han et al., 2019). In a new pandemic, uncertainty is even more ubiquitous and should be normalized, a perspective physicians may not be trained to take (Simpkin & Schwartzstein, 2016) due to societal and organizational pressure for certainty. Organizations and authorities should thus make it explicit and make useful suggestions on how to stay effective in the face of uncertainty. Facilitating access to information backed by official bodies, or even interactive training of uncertainty management are conceivable options. Taking a long-term perspective, routinely implementing uncertainty management in medical education seems called for, as has been noted before the pandemic (e.g., Tonelli & Upshur, 2019). The goal of this must be to increase tolerance of uncertainty if certainty is not available (cf. Koffman et al., 2020). As reflected in the model proposed by Hillen et al. (2017), while intolerance may lead to undesirable decision behavior, tolerance can help maintain the ability to make appropriate decisions in the face of uncertainty. Of course, the distinction between unavoidable and avoidable uncertainty is crucial for this. Otherwise, excess tolerance of uncertainty (Reis-Dennis et al., 2021) may result in blindly sticking to alleged certainties contradicting scientific evidence (Levin et al., 2022).

Structural prevention and intervention measures at the organizational level are equally called for to ensure that hospitals and other health institutions are equipped to support doctors. This claim is in line with contextual influences, e.g., cultural or social factors, as moderators of the extent to which uncertainty leads to negative or more positive appraisals and reactions (Hillen et al., 2017). The aim of such interventions would be to provide as much certainty as possible. For example, effective and transparent communication of guidelines, procedures, and protocols is crucial (Lynch et al., 2022). This pandemic has elicited a lot of unknowns. Even so, up-to-date, easy to find guidelines, regular time allotted for information and exchange, clear and stable communication channels, and avoidance of unnecessary ambiguities are examples of effective tools for preventing the situation from becoming more uncertain than it already is. Also, it is obvious that medical decision making and lack of knowledge are not the only areas of problematic uncertainty for physicians regarding COVID-19. For example, Fernemark et al. (2022) reported that uncertainty about infection risk troubled Swedish primary care physicians, which stresses the importance of providing adequate safety equipment, thus freeing staff’s capacity to handle the less avoidable uncertainties of the pandemic.

In a similar vein, especially in communication with patients or their relatives, it is important that physicians do not have to carry the burden of their own and others’ uncertainty all by themselves (Koffman et al., 2020). Providing specialized staff, such as social workers or psychologists, and encouraging exchange among professions, would help prevent placing undue burden on physicians.

## Conclusions

In this article, we presented findings of a systematic search and scoping review of research studies designed to examine the emotional and functional consequences of IU and medical decision-making uncertainty among physician populations during the COVID-19 pandemic. Findings extracted from ten articles suggested that perceptions of IU coincided with experiences of poorer mental health, particularly with regard to burnout symptoms during the pandemic period. Associations were in the small-to-medium ranges. The exact impact of IU, especially as it differs between pre- and post-pandemic periods, remains unclear. Studies designed to examine treatment decisions under COVID-19-induced uncertainty suggested that decisional uncertainties were widespread and may have facilitated the use of more aggressive or unproven treatments. Whether uncertainties indeed exerted any tangible influence on medical treatment decisions, and the magnitude of this potential influence, is yet to be established. Distress related to decision uncertainty clearly emerged as a major challenge in qualitative studies referring to the early pandemic.

Future research should continue to investigate the role of IU and uncertainty in physician mental health and medical decision making in emergency situations such as a pandemic, using best practice methods suitable to ensure the validity and reliability of findings. Reducing uncertainties in the workplace could be achieved by providing adequate safety equipment and by implementing transparent and rapid communication channels in times of pandemic disruptions. Communications should clarify any adjusted work processes and give information on the best available evidence. The presence of uncertainties should further be raised with physicians and trainees to discuss and normalize the experience of uncertainty in medical practice. While the psychological and decisional impact of uncertainty in pandemic situations warrants further research, efforts should be made to mitigate potential risks to physicians operating under high-stakes and novel situations.

## Data Availability

Not applicable.

## Footnotes

1 For consistency, we will continue to use the term IU except when specifically referring to the model.

2 Because in our view, Martínez-Sanz et al. (2020) did not use a valid IU measure, as we elaborate upon in the discussion, we only use their findings on medical treatment choices under uncertainty in COVID-19 scenarios for this review, featured in the Uncertainty and Medical Decision Making during COVID-19 section.
